# Effect of metal ions on Alzheimer's disease

**DOI:** 10.1002/brb3.2527

**Published:** 2022-02-24

**Authors:** Fan Liu, Zhuo Zhang, Lin Zhang, Ruo‐Ni Meng, Jia Gao, Ming Jin, Ming Li, Xiao‐Peng Wang

**Affiliations:** ^1^ Department of Neurology Second Hospital of Hebei Medical University Shijiazhuang Hebei China; ^2^ Department of Orthopaedic Surgery Third Hospital of Hebei Medical University Shijiazhuang Hebei China

**Keywords:** Alzheimer's disease, metal ions, mild cognitive impairment, neurological diseases, pathogenesis, the central nervous system, CNS

## Abstract

Alzheimer's disease (AD) is a degenerative disease of the nervous system. The typical pathological changes of AD are Aβ deposition, neurofibrillary tangles, neuron loss, and chronic inflammation. The balance of metal ions is essential for numerous physiological functions, especially in the central nervous system. More studies showed that metal ions participate in the development of AD. However, the involvement of metal ions in AD is controversial. Thus, we reviewed articles about the relationship between metal ions and AD and discussed some contradictory reports in order to better understand the role of metal ions in AD.

## Graphical abstract

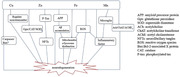

## 1 INTRODUCTION

Alzheimer's disease (AD) is a kind of dementia, of which the main clinical feature is progressive mental decline. Patients can show not only cognitive dysfunction but also have abnormal mental behavior and movement disorders (Engelhardt and Laks 2008). AD severely impairs the geriatrics quality of life and also adds great pressure to the family and society. Dementia is a global problem. The worldwide cost of dementia is increasing yearly. It is expected to reach 2.54 trillion US dollars in 2030 and 9.12 trillion US dollars in 2050 (Jia et al. 2018). This urgently requires us to devote ourselves to the research of AD with a more positive attitude.

**FIGURE 1:**
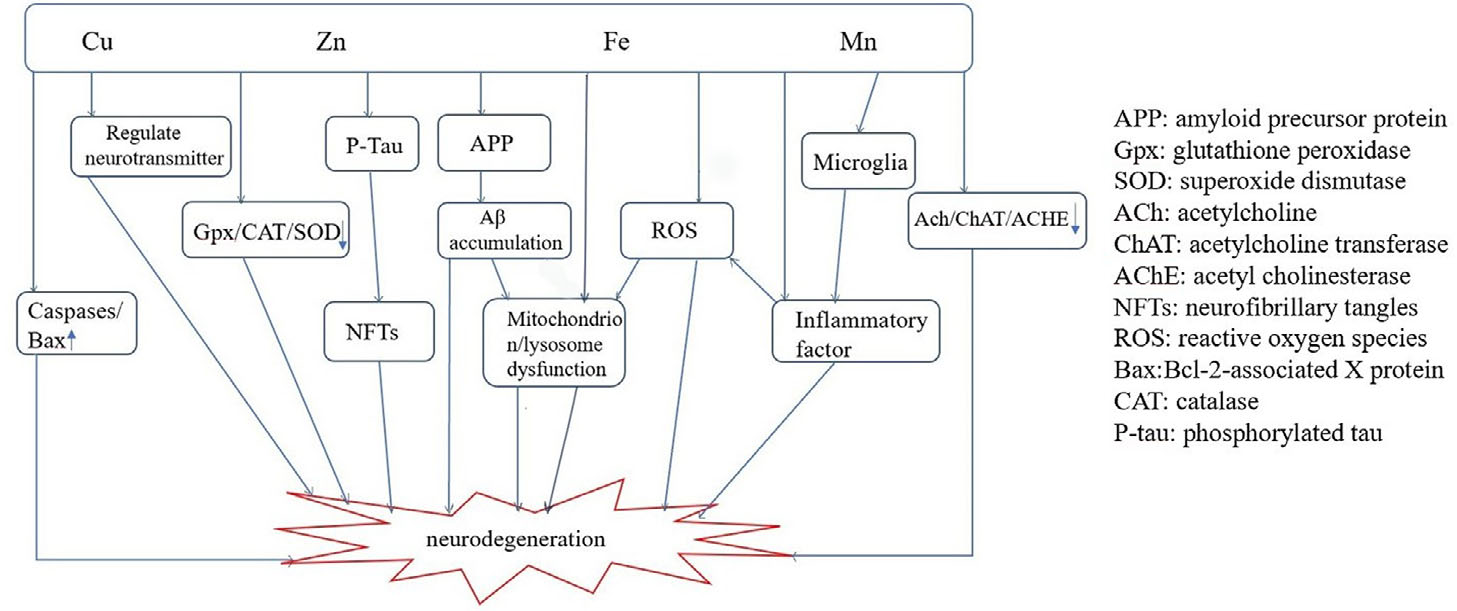
Partial mechanism of metal in AD. AD may involve multiple aspects, including Aβ metabolic disorders, Tau protein hyperphosphorylation, gene mutations, oxidative stress and free radical damage, cholinergic neuron loss, inflammatory damage, and so on. Metal ions regulate AD development by participating in these physiological processes

Within the physiological range, metal elements such as iron (Fe), zinc (Zn), copper (Cu), and manganese (Mn) play an indispensable role in body growth, metabolism, and brain development; the imbalance of metal ions is related to various diseases of the human body. In‐depth studies have found that metal ions can participate in various mechanisms related to the pathogenesis of AD, such as protein deposition, neurofibrillary tangles (NFTs), oxidative stress, neuroinflammation, and neuronal loss (Su et al. 2007) (see Figure 1). Given that AD is a disease with many risk factors and complex pathogeneses, a comprehensive understanding of the relationship between metals and AD can not only provide directions for us to take measures against the damage of metal ions before the disease but also provide targeted goals for treatment. This article reviews the current relationship between Cu ion, Fe ion, Zn ion, Mn ion, and AD.

## 2 ROLE OF METAL IONS IN AD

### 2.1 Copper

Cu is a trace element with redox activity in the human body and is widely distributed in the brain. Cu can be used as a cofactor or structural component of various enzymes, involving cell respiration, free radical detoxification, Fe metabolism, and the synthesis of neurotransmitters, neuropeptides, and hormones (Pena et al. 1999).

Ceruloplasmin is an α2 glycoprotein that has antioxidant properties. Ceruloplasmin participates in Cu transport and Fe metabolism. Ceruloplasmin is the primary form of Cu in the blood (Holmberg et al. 1948; Yang et al. 2021). In the past few years, many studies have revealed a link between the pathogenesis of AD and abnormal Cu metabolism, genetic evidence suggests that the gene controlling the Cu pathway is a susceptibility gene for AD, which has been confirmed in several studies (Bucossi et al. 2012; Squitti et al. 2021; Squitti et al. 2013). Changes in Cu levels in serum, plasma, cerebrospinal fluid (CSF), and brain are associated with the development of cognitive deficits and AD (Squitti et al. 2014). Restoration of ceruloplasmin in the AD mouse brain could reduce the damage of hippocampal neurons (Zhao et al. 2018), suggesting the neuroprotective effect of ceruloplasmin. Although most Cu in the plasma is stably bound to ceruloplasmin, some are unstable with other molecules, such as albumin and globulin. It was found that the level of non‐ceruloplasmin‐bound Cu (non‐Cp‐Cu) increased in AD and mild cognitive impairment (MCI) (Squitti et al. 2011), and it is suggested that the increase of non‐Cp‐Cu may be an indicator to predict the progression of MCI to AD. Further studies showed that non‐Cp‐Cu levels increased in the early stages of MCI. During 6 years of observation, 50% of MCI subjects with elevated non‐Cp‐Cu developed into AD patients within 4 years (Squitti et al. 2014).

β‐Amyloid (Aβ) is produced by β‐amyloid precursor protein (APP) through proteolysis of β‐ and γ‐secretase. Cu promotes the formation of Aβ plaques (Kitazawa et al. 2009). On the other hand, the Cu^2+^–Aβ complex can catalyze O_2_ to produce hydrogen peroxide (H_2_O_2_). Excessive H_2_O_2_ generates a large number of free radicals through the Fenton reaction, causing a series of lipid peroxidation, protein and DNA damage. Nguyen et al. (2015) demonstrated that bis‐8 (aminoquinoline) ligands could catalytically extract Cu^2+^ from Cu^2+^–Aβ, the Cu‐ is then fully released in the presence of glutathione, forming a Cu–glutathione complex, which is an efficient biological ligand of Cu‐ that is able to deliver Cu ions for the formation of Cu–proteins. At present, chelating agents that can specifically bind metal ions may be an important strategy for the treatment of AD (Fu et al. 2016).

Similarly, Cu can also bind to Tau proteins and promote the formation of NFTs (Bacchella et al. 2020). In addition, Tau combined with Cu shows redox activity. Tau can reduce Cu ions and promote the generation of a series of reactive oxygen species (ROS) (Su et al. 2007). Although both Tau and Aβ are critical pathological changes in AD, the exact effects of Cu and Tau on AD have not been thoroughly studied and require further investigation to find this association.

Studies have found that Cu can increase brain inflammation and promote secretion of more proinflammatory factors, such as interleukin‐1β (IL‐1β), tumor necrosis factor‐alpha (TNF‐α), and IL‐6, and downregulate the expression of LRP1 (Kitazawa et al. 2016), indicating that the inflammation promoted by Cu is one of the ways affecting the development of AD. In addition, microglia‐induced neuroinflammation is closely related to AD. Cu^2+^ can activate nuclear factor κB (NF‐κB)‐dependent microglia and produce mitochondrial ROS, and release nitric oxide (NO) and TNF‐α in a time‐ and dose‐dependent manner. The inhibition of TNF‐α or NO alone does not reduce neuronal death. Still, the combined inhibition of TNF‐α and NO could achieve this effect, so it is speculated that the combination of TNF‐α and NO could cause neuronal damage (Hu et al. 2014). The application of ROS scavengers can inhibit the neurotoxicity produced by NO and TNF‐α, indicating that the NO and TNF‐α produced by microglia and the neurotoxicity mediated by them may be related to the mitochondrial ROS‐NF‐κB signal activated by Cu^2+^ (Hu et al. 2014).

Cu is also involved in the synthesis of neurotransmitters (Spencer et al. 2011). Most previous studies have shown that Cu can inhibit glutamate receptor activity (Vlachova et al. 1996; Weiser and Wienrich 1996). Later, it was found that the regulation of synaptic function by Cu is not static but has a dual role: acute Cu exposure can inhibit the activity of N‐methyl‐d‐aspartate receptors (NMDAR) and α‐amino‐3‐hydroxy‐5‐methyl‐4‐isoxazolepropionic acid receptors (AMPAR), while chronic Cu exposure could increase the function of glutamate receptors (Bacchella et al. 2020), thus affecting learning and memory.

Cu can increase not only oxidative stress and promote the occurrence of AD by interacting with Aβ and Tau but also coregulate neural function by increasing brain inflammation and regulating synaptic function (D'Ambrosi and Rossi 2015; Hu et al. 2014; Kitazawa et al. 2016; Su et al. 2007; Spencer et al. 2011). Although the study found that Cu is closely related to Aβ and Tau pathology, the specific mechanism of action is still under further exploration.

### 2.2 Iron

Fe is a vital metal element in the brain that participates in oxygen transport and storage, cellular respiration, neurotransmitters, and DNA synthesis (Lane et al. 2018). Increased Fe was observed in the brain‐damaged area of AD patients (Maher 2018), which had a significant correlation with Aβ plaque and Tau pathology (van Duijn et al. 2017). Ferritin is a protein that stores and regulates Fe. It is related to AD. Elevated plasma and CSF ferritin levels are a feature of preclinical AD (Goozee et al. 2018). Elevated ferritin levels suggest elevated Fe levels in CSF and brain, which may be related to ferroptosis, which is a cell death pathway caused by lipid peroxide (Acevedo et al. 2019).

Intracellular Fe can regulate the translation of APP. APP mRNA encodes a functional Fe response element RNA stem‐loop, which binds to Fe regulatory proteins (Rogers et al. 2008). When intracellular Fe increases, it can upregulate the expression of APP and produce more Aβ (Becerril‐Ortega et al. 2014). Aβ1‐42 could reduce the survival rate of nerve cells, and the presence of Fe^3+^ makes this situation more serious (Nishizaki 2019). Further study found that Aβ1‐42 could activate Caspase‐3, Caspase‐4, and Caspase‐8 in varying degrees, and Fe^3+^ further enhanced the activation of Caspase‐3 and Caspase‐4 induced by Aβ1‐42, thus promoting nerve cell death (Guo et al. 2013). Excessive Fe load in the brain can also promote Tau hyperphosphorylation through cyclin‐dependent kinase 5 and glycogen synthase kinase‐3β (GSK‐3β) pathways, and then promote the formation of NFTs, while the application of deferoxamine in APP/PS1 transgenic mice can inhibit Tau phosphorylation (Tsatsanis et al. 2019). On the other hand, insufficient Fe efflux is considered one of the mechanisms of Fe metabolism disorders. APP plays a vital role in Fe homeostasis by stabilizing ferroportin that promotes intracellular Fe efflux (Belaidi et al. 2018). The brain Fe level of APP gene knockout mice significantly increases with age compared with the control group (Wong et al. 2014). Hyperphosphorylation and aggregation of Tau, in turn, impairs the transport of APP to the cell membrane, resulting in Fe accumulation in neurons (Nishizaki 2019). Finally, all of these lead to a vicious circle of Tau pathology and Fe accumulation.

Mitochondria are the crucial organelles of cellular Fe metabolism. A large amount of ROS will be produced through the Fenton and Haber–Weiss reactions when Fe is overloaded, which is the primary way for the body to produce ROS. Mitochondrial ferritin (FtMt) is a kind of ferritin accumulated in the mitochondria, which is related to Fe storage and distribution and reduces oxidative damage of mitochondria. There are high levels of FtMt mRNA and protein in the cerebral cortex of AD. Treating cells with H_2_O_2_ can increase FtMt mRNA and protein levels, thus confirming that FtMt may have a neuroprotective effect on oxidative stress (Wang et al. 2011). In addition, when using Aβ25‐35 to deal with FtMt knockout mice, its Bcl‐2/Bax ratio decreased, and the caspase‐3 level and poly ADP ribose polymerase activity and cell death increased; thus, it can be seen that FtMt deficiency can exacerbate nervous system damage caused by Aβ25‐35 (Wang et al. 2017). Mitoferrin‐1 is the main protein on the mitochondrial membrane that participates in transporting Fe from the cytoplasm to the mitochondria and is involved in regulating mitochondrial Fe. It is found that Mitoferrin‐1 regulates Fe metabolism by changing Fe levels in the mitochondria, the expression of Fe–sulfur protein and ferritin‐related genes in the *Caenorhabditis elegans* model of AD. Knockdown of mitoferrin‐1 could reduce mitochondrial Fe content and reduce the level of mitochondrial ROS, and at the same time, Aβ reduction is also observed in the model (Huang et al. 2018). This shows that Mitoferrin‐1 is important in developing AD by affecting Fe metabolism and interfering with mitochondrial function. Mitoferrin‐1 is expected to be a new direction of research in AD.

Ferroptosis is a new mode of death first reported in 2012 (Conrad and Friedmann Angeli 2015), which is closely related to various diseases (Fanzani and Poli 2017). Ferroptosis is characterized by the accumulation of Fe‐dependent ROS, the decrease of glutathione (GSH) levels, and the inactivation of glutathione peroxidase 4 (GPX4). The latest study found that ferroptosis suppressor protein 1 (FSP1) plays a similar role to GPX4 in the process of ferroptosis, but it can prevent lipid peroxidation and ferroptosis independently of the GPX4 and GSH pathways, which may be related to FSP1‐coenzyme Q10 (CoQ10)‐nicotinamide adenine dinucleotide phosphate (NADPH) signaling pathway (Doll et al. 2019). Intracellular Fe accumulation can produce ROS through the Fenton reaction, which can cause oxidative damage of many vital proteins and trigger a variety of apoptotic signal pathways (Maher 2018). Lipoxygenase belongs to oxidoreductase, which can catalyze the production of various lipid hydroperoxides from polyunsaturated fatty acids, thereby changing the permeability and integrity of the membrane and promoting the occurrence of ferroptosis. The active site of lipoxygenase is closely related to the Fe ion. Fe chelating agents can reduce lipid peroxidation and ferroptosis. GPX4 is an important antiperoxidase. In the process of ferroptosis, lipid peroxidation caused by Fe will cause catastrophic membrane rupture when the activity of GPX4 is reduced (Dixon 2012). GSH is an antioxidant closely related to the role of GPX4. Studies have found that Fe may specifically promote cell death when the level of GSH in vivo is reduced, although, at these concentrations, Fe itself is not toxic (van Duijn et al. 2017).

From another point of view, the above studies suggest the application prospect of Fe chelating agents in AD treatment. However, there are still many problems to be solved in practical work, such as improving the ability of metal chelating agents to pass through the blood–brain barrier, enhancing the accuracy of Fe chelating agents, and reducing excess Fe without affecting the normal physiological function of metals, so the clinical application of Fe chelating agents still needs to be optimized.

### 2.3 Zinc

Zn is the second abundant trace element in human body after Fe. A meta‐analysis based on all relevant studies published between 1984 and 2014 showed a significant reduction in serum Zn levels in patients with AD (Wang 2015), and the increase in Zn content in the cerebral cortex is related to Aβ pathology and the severity of dementia (Religa et al. 2006). With the continuous development of imaging technology, there are more imaging studies on AD. Positron emission tomography imaging found a clear defect in the clearance of Zn ions in brain regions associated with AD cognitive impairment (DeGrado et al. 2016).

Lovell et al. (1998) reported that a comparison of AD and control neuropil revealed a significant (*p*  <  .05) elevation of Zn in AD subjects and observed that Cu, Fe, and particularly Zn could accelerate the aggregation of Aβ‐peptide (Lovell et al. 1998). Studies using Zn supplementation as a treatment for AD found that dietary Zn supplementation can reduce Aβ, Tau pathology, and cognitive impairment in the hippocampus of AD transgenic mice and increase the level of brain‐derived neurotrophic factor (BDNF) in mouse models (Corona et al. 2010). It is well known that Aβ is produced by APP being cleaved by β‐secretase and γ‐secretase, and APP is cut by an enzyme with α‐secretase activity, which produces the N‐terminal fragment sAPPα, which is an effective neurotrophic factor. Zn treatment can reduce Tau phosphorylation and GSK‐3β levels in PC12 cells induced by Aβ and reduce Aβ by lowering the activity of γ‐secretase (Li et al. 2018). This again proves the neuroprotective effect of Zn. However, some study show that high‐dose zinc treatment will increases the level of APP and the activity of β‐secretase, resulting in increased secretion of sAPPβ over sAPPα in the transgenic mouse brain. All of these changes promote the pathology of Aβ (Wang et al. 2010). After using Zn‐containing nanoparticles to increase Zn levels in the brains of AD mice, Aβ and proinflammatory factors were significantly reduced; increased brain Zn might be beneficial rescuing some pathological alterations caused by Zn deficiency (Vilella et al. 2018). As we all know, Aβ and Tau pathology are essential changes in the pathogenesis of AD. Studies have found that glutamate neuron excitation‐mediated Zn release increases Tau pathology, which may be due to Zn inhibiting the activity of protein phosphatase 2A (Sun et al. 2012). This may also explain why Tau pathology tends to develop in the area where glutamatergic neurons are abundant. Liang and coworkers (Hu et al. 2017) established an inducible cell model and found that Zn ions can promote the accumulation of Tau protein and produce cytotoxicity. This is because the combination of Zn ions with specific amino acids of full‐length Tau protein makes it a more easily accumulated structure, and this accumulation can further amplify Tau protein toxicity by inducing endogenous Tau protein accumulation and abnormal phosphorylation. Excessive Zn supplementation can promote Tau protein phosphorylation and cognitive impairment in mice containing human Tau protein genes, which further confirms the effect of Zn on Tau pathology (Craven et al. 2018).

Apolipoprotein E (APOE) is a vital protein in the human body. E2, E3, and E4 are three known alleles. Among them, the genetic variation of APOE4 is the most important genetic risk factor for AD, which greatly increases the incidence of AD. The presence of APOE4 makes human neurons more susceptible to toxic damage and can promote brain atrophy, Tau pathology, and Aβ deposition (Wadhwani et al. 2019), which also explains the phenomenon of cognitive decline in patients carrying the APOE4 gene (Lin et al. 2016). The study found that APOE4 synergizes with Zn ions by activating the Erk pathway, increasing the degree of Tau phosphorylation in mice and neuronal cells, suggesting that reducing Zn concentrations may be beneficial for reducing the pathology of patients with APOE4 gene expression (Harris et al. 2004). Zn can increase plaque deposition by promoting the accumulation of APOE/Aβ complexes. The presence of a large amount of Zn in or around the APOE/Aβ complex may reduce the activity of degrading Aβ protease or hinder its complete contact with Aβ to function. Consumption of Zn can improve the ability of protease to degrade Aβ (Oh et al. 2020). Like Cu, Zn is also involved in the process of neurotransmission. Glutamate neurons release Zn and glutamate at the same time. Zn ions interact with various ion channels and post‐synaptic membrane receptors to regulate synaptic plasticity and affect learning and memory functions and behavioral activities. The excessive release of glutamate can excessively activate NMDAR and trigger the opening of related ion channels, promote a significant increase in intracellular calcium and trigger apoptosis (Arundine and Tymianski 2003). The autopsy results of an AD patient showed that the level of Zn released from hippocampal synaptic vesicles was about three times that of the control group (Bjorklund et al. 2012). In vitro studies have shown that Zn has a bidirectional effect on extracellular glutamate levels. Glutamate levels were promoted at low Zn concentrations and inhibited at high Zn concentrations. After applying glutamate uptake blockers, high concentrations of Zn can also promote the release of glutamate. Further research indicates that calcium/calmodulin‐dependent protein kinase II (CaMKII) may be related to the mechanism of Zn ions promoting glutamate release, and the excitatory amino acid transporter may be related to the mechanism of Zn reducing glutamate levels (Shen et al. 2020).

Neuronal loss is an important feature of AD. The excessive accumulation of Zn in cells can not only change the permeability of lysosomes but also hinder mitochondrial energy production and activate mitochondrial permeability transition pores to increase the generation and release of the apoptosis factor, thereby mediating neuronal death (Jiang et al. 2001). Splicing factor proline and glutamine‐rich (SFPQ) is widely present in the nucleus of animal cells, and its expression is most evident in the cerebral cortex and hippocampus. It is closely related to transcription, DNA repair, neuronal differentiation, and development. The disorder of SFPQ was found in AD and frontotemporal lobar dementia. The nuclear‐cytoplasmic distribution balance of SFPQ is vital in maintaining the homeostasis of cells and responding to various stimuli. A high concentration of Zn in cells can induce SFPQ to accumulate in the cytoplasm, resulting in abnormal gene regulation (Huang et al. 2020). The disorder of SFPQ and the subsequent disintegration of DNA and abnormal transcription may be a new way for AD to occur (Lu et al. 2018).

At present, serum Zn was significantly decreased in AD patients (Li et al. 2017; Ventriglia et al. 2015). The relationship between Zn and the onset of AD still needs further study, but in any case, strict control of Zn content in the body is necessary.

### 2.4 Manganese

Mn is a necessary nutrient element to maintain the physiological functions of the human body. In the central nervous system (CNS), Mn is an essential cofactor for several enzymes, including DNA and RNA polymerases, peptidases, carboxylases, superoxide dismutase (SOD), and glutamine synthetase (GS) (Aschner et al. 2007; Reddi et al. 2009). A meta‐study found that the serum Mn levels are lower in AD patients, and Mn deficiency may be a risk factor for AD (Du et al. 2017), showing some kind of connection between Mn and AD. However, various organs will be damaged after excess Mn exposure, especially the CNS, resulting in a neurodegenerative disease affecting cortical structures and basal ganglia (Dobson et al. 2004).

The cholinergic theory is widely studied, and currently, there are drugs for the treatment of AD according to it. The basal forebrain is a crucial central cholinergic region, establishing the SN56 cells as the basal forebrain cholinergic neuron model to study the toxicity mechanism of neurons after Mn exposure. The study found that after acute and long‐term Mn exposure, acetylcholine levels decreased, acetylcholine transferase activity decreased and acetylcholinesterase activity increased. It is well known that BDNF can promote the survival of cholinergic neurons, which is closely related to synaptic plasticity and affect people's learning and memory functions. However, Mn exposure can reduce BDNF expression in the rat hippocampus (Wang et al. 2017). A population study found that occupational Mn exposure reduced the plasma BDNF and cognitive ability of the population, and the degree of BDNF decline was positively correlated with the degree of cognitive impairment (Zou et al. 2014).

It is well known that NF‐κB is related to the activation of glial cells and the production of inflammatory factors (Kirkley et al. 2017). Glial cells are the main target of Mn. Mn can increase the number of inflammatory factors produced by NF‐κB‐regulated microglia and astrocytes (Chen et al. 2006; Spranger et al. 1998). Inhibiting NF‐κB in glial cells has anti‐inflammatory and neuroprotective effects. In‐depth studies have found that the expression of inflammatory genes regulated by NF‐κB in Mn‐treated mixed glial cells (microglia and astrocytes) is significantly higher than in single microglia or astrocytes. The survival rate of neurons in mixed glial cell culture fluid exposed to Mn was significantly lower than in single glial cell culture fluid. This indicates that the interaction between microglia and astrocytes can produce more destructive inflammatory mediators and enhance the neuroinflammatory damage caused by Mn exposure (Popichak et al. 2018).

Mn blocks APP and heavy‐chain ferritin protein translation in a dose‐ and time‐dependent manner, leading to the accumulation of Fe^2+^. Increasing APP expression can partially reduce Mn‐induced ROS production and neurotoxicity (Rogers et al. 2019; Venkataramani et al. 2018). On the other hand, Mn can also weaken the body's antioxidant defense. Mn treatment significantly increased intracellular ROS and malondialdehyde levels, while GSH levels, SOD, and GPX4 activity were significantly reduced. Antioxidants applied to Mn‐treated cells were able to reverse these results (Bahar et al. 2017). The increase of Mn concentration initially promoted the increase of oxidative stress. The increased oxidative stress in the body can further lead to the imbalance of Fe^3+^ and Fe^2+^ homeostasis, which in turn induces a variety of Fe‐mediated neuronal damage mechanisms, thereby exaggerating the Mn‐induced neurodegeneration (Fernsebner et al. 2014).

Astrocytes are the most abundant glial cells in the brain and are vital for normal brain function, one of the functions of astrocytes is to regulate synaptic activity and maintain glutamate levels. Glutamate levels are increased by the accumulation of Mn in the brain (Fernsebner et al. 2014). This may be due to the GS associated with GS in astrocytes, and Mn inhibits the glutamate transporter related to glutamate uptake. All of these results in elevated glutamate levels that mediate neuroexcitatory toxicity have been shown to be connected with various neurodegenerative diseases (Deng et al. 2012; Lee et al. 2017).

The Mn pollution in the environment is becoming more serious. Although MN is an essential metal element for the human body, too much Mn exposure can disrupt normal nerve function and participate in AD through neuroinflammation, oxidative stress, neuronal loss, and regulation of neurotransmitters. The mechanism of this has yet to be further verified.

## 3 CONCLUSIONS

The current study found that the pathogenesis of AD may involve multiple aspects, including Aβ metabolic disorders, Tau protein hyperphosphorylation, gene mutations, oxidative stress and free radical damage, cholinergic neuron loss, inflammatory damage, and so on. It is also because of the multiple pathways of AD pathogenesis that it is challenging to develop drugs for AD. Existing drugs can only improve symptoms to a certain extent, and there is a lack of drugs that can prevent the disease process or reverse its pathophysiological process. A single factor does not cause AD. It is very challenging to design drugs that target multiple areas without losing their specificity. A detailed understanding of its physiological regulation process, cellular and molecular mechanisms, and its changes in AD may be able to provide help for precision treatment. Metal‐containing protein may be beneficial or harmful. How to adjust the balance needs to be noticed in metal chelator research. Since the potential dangers of metals are known, how can they be prevented in daily life? With the continuous improvement of our understanding of AD, our treatment should be more targeted. It may be more beneficial to choose different disease stages of AD patients or even distinguish specific types and stages of metal imbalance patients.

## CONFLICT OF INTEREST

The authors report no conflict of interest.

## AUTHOR CONTRIBUTIONS

F. L. have made substantial contributions to conception and design; Z. Z., L. Z., R. N. M., and J. G. worked on the acquisition, analysis, and interpretation of data; F. L., M. J., M. L., and X. P. W. have been involved in drafting the manuscript and revising it critically for important intellectual content; X. P. W. have given final approval of the version to be published.

## CONSENT FOR PUBLICATION

The manuscript is not submitted for publication or consideration elsewhere.

### PEER REVIEW

The peer review history for this article is available at https://publons.com/publon/10.1002/brb3.2527

## Data Availability

All data generated or analyzed during this study are included in this published article.
